# Synergetic strategies for carbon neutrality and clean air

**DOI:** 10.1016/j.ese.2024.100497

**Published:** 2024-10-15

**Authors:** Steven J. Davis

**Affiliations:** Department of Earth System Science, Stanford University, Stanford, CA, 94305, United States

## Abstract

• A systematic analysis framework was proposed by Lei et al., 2024.
• China's shows promising progress towards carbon neutrality and clean air.
• China's synergetic roadmap could serve as a model for other countries.

## Graphical abstract

**Figure:**
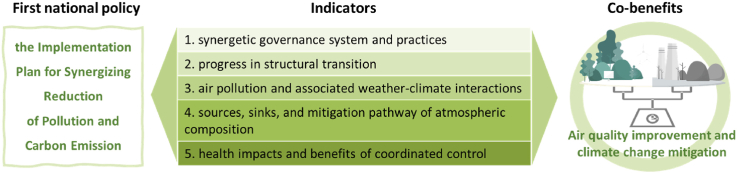

Human activities, including the burning of fossil fuels, industrial production, transportation, residential, etc., are the main sources of both air pollution and greenhouse gas (GHG) emissions. Thus, the same efforts may at once improve air quality and help to avoid climate change, and it is a research priority to investigate which interventions are most cost-effective and at what scale to meet both environmental goals [[1], [2], [3]].

During its rapid economic development in the early 2000s, China's air pollution and GHG emissions surged, threatening both public health and long-term economic stability [4,5]. But although China remains the world's largest CO_2_ emitter, accounting for about 30% of global CO_2_ emissions in 2019 (International Energy Agency: World Energy Outlook 2020), Lei et al. (2024) [6] report that the average PM_2.5_ concentration in 2021 was 35% lower than 2015 levels in 339 cities, with the ratio of severely polluted days across the country simultaneously decreasing by 58%. Such progress deserves to be celebrated, but further improvements are possible: roughly one-third of the same cities still experienced PM_2.5_ concentrations exceeding China's National Air Quality Standard, and all the cities' PM_2.5_ concentrations significantly exceeded the stricter WHO guideline (i.e., 5 μg m^−3^). For context, the average annual PM_2.5_ concentrations in the United States and the European Union were 9.1 and 10.4 μg m^−3^, respectively, in 2023, considerably lower than the 32.5 μg m^−3^ in China the same year; while in India, the annual average PM_2.5_ concentration was 54.4 μg m^−3^ in 2023 (https://www.iqair.com/dl/2023_World_Air_Quality_Report.pdf).

Chinese policymakers are thus exploring options that could deliver both further air quality improvements and contribute toward the country's Carbon Neutrality commitment, such as the Implementation Plan for Synergizing Reduction of Pollution and Carbon Emission promulgated in June 2022. Timely scientific assessments of progress such as that of Lei et al. (2024) [6] are critical to inform the implementation of such policies (see Fig. 1). The authors analyze the actions that have been taken and their consequences, evaluating 20 different indicators across 5 dimensions: (1) synergetic governance system and practices, (2) progress in structural transition, (3) air pollution and associated weather-climate interactions, (4) sources, sinks, and mitigation pathway of atmospheric composition, and (5) health impacts and benefits of coordinated control.

**Fig. 1:**
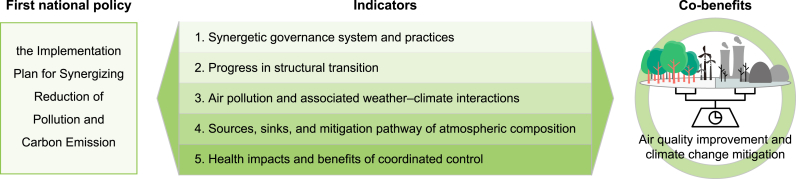
The Implementation Plan for Synergizing Reduction of Pollution and Carbon Emission policy is aimed at acheiving both air quality improvements and climate change mitigation. Lei et al. assess a number of indicators of the policy's effectiveness.

Their findings reveal both key milestones and future governance challenges for policymakers. For example, China has been the dominant global consumer of coal for decades, but by the end of 2021, coal-fired power units with a total capacity of more than one thousand GW had achieved ultralow emission standards, thereby establishing the largest clean coal-fired power generation system globally. The production of crude steel and cement declined after years of growth, representing a decrease in demand from energy-intensive industries like the construction sector. But perhaps most impressively, nearly half (47%) of Chinese electricity in 2021 was generated from non-fossil energy sources, indicating that a remarkable change in the structure of the country's energy systems is underway. Such robust expansion of clean energy facilitates further improvements in the transportation sector, with the penetration rate of new energy vehicles surging to 13.4%.

However, Lei et al. (2024) [6] also find unrealized opportunities for synergistic reduction of pollutant and carbon emissions among cities and provinces, especially during the period of 2015–2020. Roughly two-thirds of cities did not achieve a co-reduction in PM_2.5_ concentrations and CO_2_ emissions during that period, and 22 of the 33 provinces experienced an increase in both—mainly due to changes in the electricity and heating sector. End-of-pipe management has in many cases suppressed the rise in PM_2.5_ levels, but increases in CO_2_ emissions underscores the potential to shift focus towards technologies and systems that can reduce both PM_2.5_ concentrations and carbon emissions. Further increasing electricity generation from clean, renewable resources [7], replacing high-carbon industrial sources with lower-carbon alternatives [8], and storing and capturing carbon [9] can all contribute to both air quality and climate goals.

China is the first country to actively promote air pollutants and carbon co-control by implementing a national policy that weighs the two sides equally. Unlike developed countries that confronted regional air pollution long before committing to global climate goals, emerging markets today are facing the two issues in parallel. For instance, as early as 70 years ago, countries such as the United Kingdom and the United States began implementing national legislation to cut air pollutant emissions from sources such as industrial facilities and vehicles, which made the air pollution level reaching a low level when these countries started seriously tackling climate change issues. Given this timing, it is almost inevitable that these developing countries will seek to maximize the air quality and climate benefits through co-management of carbon and pollutants policy packages [10,11]. China's approach could therefore serve as a model for other countries seeking to reconcile environmental protection with economic development.

## Declaration of competing interest

The authors declare that they have no known competing financial interests or personal relationships that could have appeared to influence the work reported in this paper.
